# First-Complete Excision of the Stomach in a Human Being

**Published:** 1898-02

**Authors:** 


### FIRST COMPLETE EXCISION OF THE STOMACH IN A HUMAN BEING.

Dr. Carl Schlatter, in the *Medical Record* of Dec. 25, 1897, describes the case as follows: The personal observation forming the subject of this paper relates to a woman 56 years old. In her case I completely excised the stomach, even beyond its cardiac extremity, and then restored the continuity of the alimentary canal by stitching a loop of small intestine into the lower end of the esophagus, *i. e.*, esophago-enterostomy. I first saw the patient at the surgical polyclinic on Aug. 26, 1897. An inspection of the abdomen revealed a marked bulging between the left hypochondriac region and the umbilicus. The abdominal parietes were flabby, and palpation easily revealed an oval mass of hard consistency in the region of the stomach. The tumor was freely movable. Its size was about that of two fists. Very marked emaciation was found. The patient was unable to retain any kind of nourishment. She clamored for relief by surgical interference. She was admitted to my wards for further careful observation. I did not feel confident that gastrectomy, or even gastro-enterostomy, could be successfully performed, on account of the large size of the tumor. The patient continued to reject almost everything, including fluids. The iodid reaction of her saliva (after exhibition of iodid of potassium) required forty-seven minutes for its first appearance. The chemic examination of her gastric secretion showed no trace of free hydrochloric acid. . . . On Sept. 6, 1897, acting for Professor Kronlein, I performed laparotomy under morphin-ether anesthesia, and with strict antisepsis, incision in the median line, extending from the ensiform process to the umbilicus. As I had anticipated, the entire stomach presented itself in the shape of a hard mass extending from the cardiac to the pyloric extremity. Strangely enough, the tumor was freely movable. It was readily lifted out of the peritoneal cavity. Three rather soft lymph nodes were found at the greater curvature near the pylorus. The stomach being diseased *in toto*, a gastro-enterostomy was impossible. I at once decided to attempt to excise the entire organ, or take recourse in a jejunostomy. I first freed the stomach from all its attachments at the greater and lesser curvature, having previously shut off the general cavity of the peritoneum by sterilized compresses. The omentum was incised between Pean's forceps. Silk sutures were used. The stomach was then forcibly dragged downward so as to enable me to reach the esophagus. The left lobe of the liver had to be constantly held upward by an assistant, in order to permit me freely to manipulate within the field of operation. In this way I finally succeeded in securing the esophagus rather high up, by means of a Wolfler clamp. A Stille forceps was next fastened closely to the cardiac end of the tumor. Then the stomach was severed directly beneath the esophageal extremity. As the esophageal incision appeared somewhat oblique, I proceeded to place a small occluding suture at the gastric wound. The same steps were now repeated at the pyloric end of the stomach. I next mobilized the deodenum as far as possible toward the head of the pancreas. Then having applied a duodenal compressor, and likewise a tumor clamp, I removed the entire stomach between the two points of compression. I also dissected out the lymphatic nodes above mentioned. The patent lumen of the duodenum was treated, like the esophageal opening, with iodoform gauze. The broad bridge joining together different divisions of the alimentary canal had now been entirely removed. I next tried to pull the duodenal opening upward toward the esophageal cleft. It was only with considerable difficulty that the two could be made to touch. It was manifestly impossible to join them by direct suture. I therefore invaginated the duodenal rim and closed the opening by a double suture. I then searched for a suitable coil of small intestine. Beginning at the duodeno-jejunal fold, I followed down the intestine for about fifteen inches. The presenting knuckle of intestine I grasped, and pulling it over the transverse colon, I placed it against the esophageal slit. A piece of this intestine, about five inches in length, was secured between two Wolfler clamps. By means of sutures not going deeper than the serous coat, the intestine was then attached to the esophageal stump. A longitudinal slit about one inch in length was then made into the bowel. Then the mucous membrane of the esophageal end was firmly united with the intestinal mucous membrane by a continuous circular suture, The material employed was silk. Above this a second suture, extending through the muscular and serous coats, was introduced. A Lembert suture finally completed the stitching, which now seemed to hold. The esophageal and duodenal clamps were then removed, the former having remained in position for over two hours. On dropping back the organs into the abdominal cavity, the sutured portions showed marked retraction upward toward the esophageal part of the diaphragm. The abdominal wound was closed in the ordinary way by silk ligatures. Less than eight ounces of ether had been employed during the narcosis, which had fortunately been a very quiet one. Pulse after the operation 96 a minute, steady and of fair volume. There had been a very slight loss of blood during the course of the operation, which, however, had lasted nearly two hours and a half. There was a steadily progressive increase in the weight of the patient after removal of the cancerous stomach. . . . There being no food receptacle after ablation of the stomach, it became obligatory to feed my patient at first with minute quantities of food, given at short intervals. The results of this method of procedure were in all respects happy ones. Quantities of food approaching ten ounces seemed to excite vomiting. So, too, cold fluids resulted in diarrheal discharges, and may have been partly responsible for the rise in temperature, observed for some little time after the operation. Keeping in mind the absence of mechanical functions, the patient's dietary was at first a strictly fluid one. But as early as the second week after removal of the stomach, semi-solid and even solid food was allowed. It was retained and digested without discomfort. The patient having only a single tooth, mastication was of course quite imperfect, otherwise it seems to me possible that an ordinary mixed diet might have succeeded at a still earlier date. Some weeks after the operation the patient's ordinary daily dietary was as follows: At regular intervals of from two to three hours she took milk, eggs, thin gruel or pap, tea, meat, rolls, butter and Malaga wine. The daily quantity amounted to one quart of milk, two eggs, two to three onnces of pap or gruel, seven ounces of meat, seven ounces of oatmeal or barley water (as thick almost as gruel), one cup of tea, two rolls and half an ounce of butter. Personally I felt most concerned about the obliteration of all chemic activity on the part of the absent stomach.

I soon perceived that adding pepsin and hydrochloric acid to the food was theoretically as inadmissible as it had been found practically valueless. The alkaline fluids of the intestine at once neutralized the acid and rendered the pepsin inert. Fortunately it soon became apparent that despite the absence of acid pepsin, proteids were readily assimilated in the intestinal tract. . . . Products of abnormal intestinal fermentation or decomposition (skatoxyl and indoxyl) were either not at all found, or else discovered only in traces. . . . The patient objected to swallowing charcoal. Huckleberries were at three different times found in the passages, twenty-four hours after having been swallowed. Apart from a daily recurring diminution in the quantity of excreted chlorids, the urine of this woman has remained normal since ablation of her stomach. The daily excretion of chlorid of sodium has been found to vary between the limits of 0.6 per cent. and 0.95 per cent. It should be stated in this connection, however, that, complying with the wish of the patient, her food is prepared with less salt than that of the other ward patients. The stools were well formed, of normal consistency, and light yellow in color. The microscope showed large numbers of fat globules and fatty crystals, some undigested vegetable fibers, but no undigested animal fibers or connective tissue. Large quantities of triple phosphates were observed. The number of micro-organisms was normal. Altogether, repeated examinations revealed no noteworthy departure from a condition of perfect health. No matter what theoretic physiologic notions we may have imbibed from lectures and text-books, the woman under observation had repeated attacks of ordinary nausea, retching and vomiting. We must needs conclude, therefore, that the role of the stomach (*i. e.*, its antiperistaltic efficacy) in this direction has been very much overrated. While the vomited substances showed an acid reaction, this was not due to the presence of free hydrochloric acid. In view of the fact that the patient ejected as much as thirty ounces at one time, it seems reasonable to suppose that the remaining portion of the duodenum may have already begun to show distention sufficient to produce a sort of compensatory receptacle for food, perhaps nature's attempt in the direction of the new formation of a stomach. In endeavoring to explain vomiting without a stomach we should remember that the act itself is far from being a simple process. It is due to nervous action on a complex motor apparatus, consisting of pharynx, esophagus, stomach, diaphragm and abdominal muscles. It is not surprising, therefore, to have witnessed in this woman an ordinary attack of bilious vomiting, superinduced by a mere psychic disturbance.—*Abstract.*—*The Journal.*

